# Study on Axial Dispersion Characteristics of Double-Layer Prefabricated Fragments

**DOI:** 10.3390/ma16113966

**Published:** 2023-05-25

**Authors:** Yuan He, Lei Guo, Chuanting Wang, Jinyi Du, Heng Wang, Yong He

**Affiliations:** 1 School of Mechanical Engineering, Nanjing University of Science & Technology, Nanjing 210094, China; 2 Xi’an Institute of Modern Control Technology, Xi’an 710065, China

**Keywords:** three-stage detonation driving model, double-layer prefabricated fragments, fragment initial parameters, explosion detonation

## Abstract

The axial distribution of initial velocity and direction angle of double-layer prefabricated fragments after an explosion were investigated via an explosion detonation test. A three-stage detonation driving model of double-layer prefabricated fragments was proposed. In the three-stage driving model, the acceleration process of double-layer prefabricated fragments is divided into three stages: “detonation wave acceleration stage”, “metal–medium interaction stage” and “detonation products acceleration stage”. The initial parameters of each layer of prefabricated fragments calculated by the three-stage detonation driving model of double-layer prefabricated fragments fit well with the test results. It was shown that the energy utilization rate of detonation products acting on the inner-layer and outer-layer fragments were 69% and 56%, respectively. The deceleration effect of sparse waves on the outer layer of fragments was weaker than that on the inner layer. The maximum initial velocity of fragments was located near the center of the warhead where the sparse waves intersected, located at around 0.66 times of the full length of warhead. This model can provide theoretical support and a design scheme for the initial parameter design of double-layer prefabricated fragment warheads.

## 1. Introduction

Blasting-fragmentation warheads destroy targets by the shock wave and fragments formed during the detonation. With the development of blast-fragmentation warhead technology, prefabricated fragments are often used to increase the power of the warhead. It is found that the prefabricated fragments have a high energy utilization rate and a strong killing ability towards long-distance targets. To improve the energy utilization rate of explosives, double-layer prefabricated fragments are designed to increase the number of fragments. Therefore, it is important to carry out the theoretical study of the fragments’ initial parameters, including the initial velocity and the initial direction angle [1,2,3].

The research on the initial state of fragments mainly focuses on the initial velocity and flying direction angle of fragments. Currently, studies are mainly focused on three aspects: experimental research, finite element simulation calculation, and theoretical calculation.

In experimental research, various technical means are often used to record the spatiotemporal parameters of prefabricated fragments, such as fragment initial velocity, fragment dispersion direction angle, fragment mass, etc. The commonly used technical methods include X-ray photography, high-speed photography, etc. These methods can simultaneously record data such as initial velocity of fragmentation, and direction of fragmentation. However, as research progresses, researchers have gradually discovered that for the study of initial parameters of fragments, a large amount of experimental data is required to obtain universal rules. Shortcomings such as high experimental research costs, long cycle, a lack of clearly targeted research objectives, and poor applicability of research methods have gradually emerged [4,5].

With the development of finite element simulation technology, a large number of researchers have carried out finite element simulation studies on initial parameters of fragments that can generate a large amount of data in a relatively short period of time. However, researchers have found that finite element simulation methods have very strict requirements for material constitutive models and calculation parameter settings, and even slight differences in parameters can cause differences in results. Moreover, if there are minor changes in the structure of the warhead, the finite element simulation results are unreliable, resulting in a significant waste of time and cost. These issues have led researchers to shift their focus towards more universal and efficient calculation methods [6,7].

The earliest theoretical calculation methods for the initial parameters of fragments were proposed by Gunny and Taylor, who proposed the Gunny formula for calculating the initial velocity of fragments and the Taylor formula for calculating the dispersion direction angle of fragments, respectively [8]. Based on this initial work, later scholars conducted much theoretical, experimental, and simulation work and devised various modification formulas based on the above two formulas. Some started with the axial position of fragments and proposed improved methods for calculating the axial distribution of initial velocity of fragments. Other researchers started with lateral sparse waves and added a correction term to the Taylor angle [9,10].

When the warhead contains a large number of prefabricated fragments placed in multiple layers, there are strong collisions between these prefabricated fragments during the detonation drive process, ultimately leading to changes in the fragmentation behavior. However, such a theoretical model that considers multi-layer prefabricated fragments has not yet been well developed.

Therefore, the main objective of the work was to introduce the “metal–medium interaction stage” between the “detonation wave acceleration stage” and the “detonation products acceleration stage”.

## 2. Experimental Procedures

**Figure 1:**
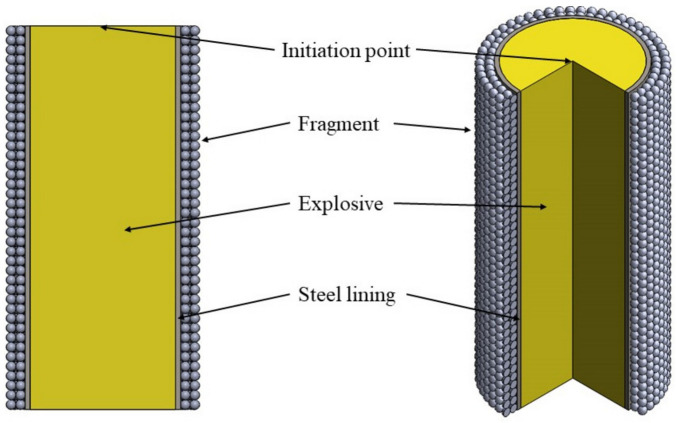
The scheme of the warhead used in the test.

The structure of the warhead for the static explosion test is shown in Figure 1. A double-layer of prefabricated fragments was placed outside the steel cylinder, the fragments were installed individually to ensure that each fragment of the outer layer was on the same radial direction as that of the inner layer. Figure 1 shows the symmetrical structure of the warhead, with the yellow part being the charge and the outer lining of the charge. The inner lining separates two layers of spherical prefabricated fragments from the explosive, with air outside the prefabricated fragments.

**Figure 2:**
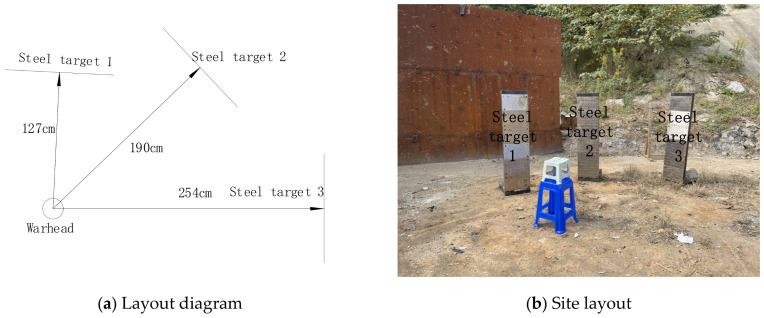
The layout of the explosion experiment.

In Figure 1, the charge radius of the warhead is 38 mm, the charge length is 100 mm, the mass of the explosive is around 760 g, the thickness of steel lining is 1.3 mm, the diameter of prefabricated tungsten fragments is 2.5 mm, and the total mass of prefabricated fragments is 806 g. The test layout is shown in Figure 2.

Prior to ignition, the warhead was placed vertically on a stand with height of 67 cm. Three steel targets were placed at various distances perpendicular to the direction of the warhead. A detonator was connected to the upper surface of the warhead charge. Each steel target center was arranged at a horizontal distance of 127 cm, 190 cm, and 254 cm. Each steel target was 150 cm high, 40 cm wide, and 1 cm thick and fixed on the target frame. In the detonation test, the fragments impacted the steel targets and formed dimples on the targets. The position of each dimple was recorded carefully to study the dispersion distribution behavior of the fragments. The diameter of each dimple was recorded carefully to calculate the corresponding depth, and these depth values were then employed to calculate the impact velocity of the fragments.

**Figure 3:**
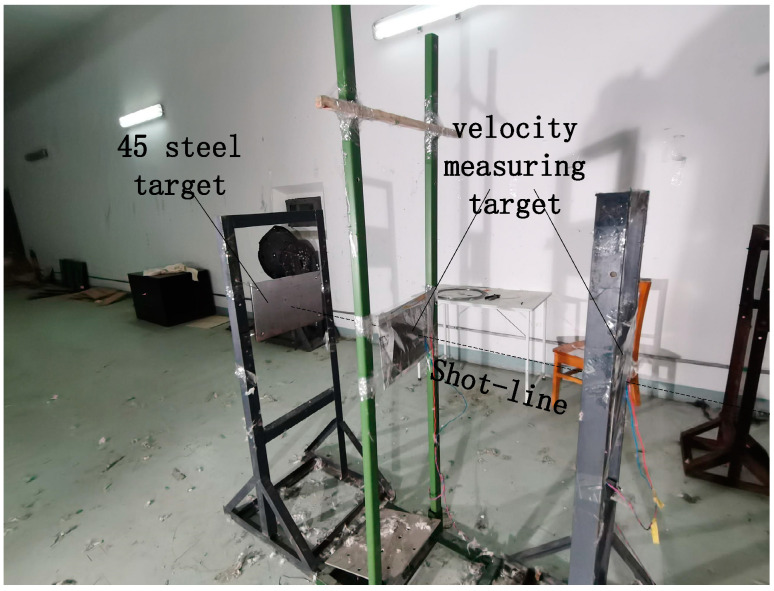
The set-up of the ballistic gun test.

The relationship between the penetration depth on a steel target and the velocity of a tungsten fragment was established by ballistic gun test. The test layout is shown in Figure 3. A ballistic gun was employed to shoot the tungsten fragments with diameter of 2.5 mm towards a steel target with thickness of 10 mm at various velocities. The velocities of the tungsten fragments were controlled by adjusting the amount of gun powder, and the velocity was measured by velocity measuring systems positioned in front of the steel target.

## 3. Experimental Results

**Figure 4:**
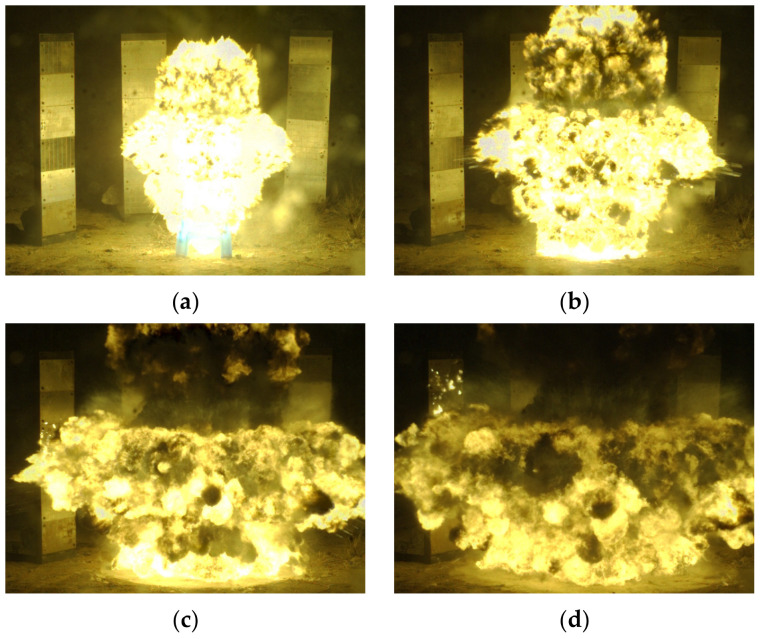
High-speed photography images of the explosion process, at (**a**) 166 µs, (**b**) 332 µs, (**c**) 664 µs and (**d**) 830 µs after ignition.

The warhead explosion process is shown in Figure 4. After ignition, the explosion expanded inside the charge and drove the fragments with high velocity. The fragments in the middle area of the cylinder warhead had the highest velocity, while the fragments at the end area had lower velocity due to the leakage effect.

**Figure 5:**
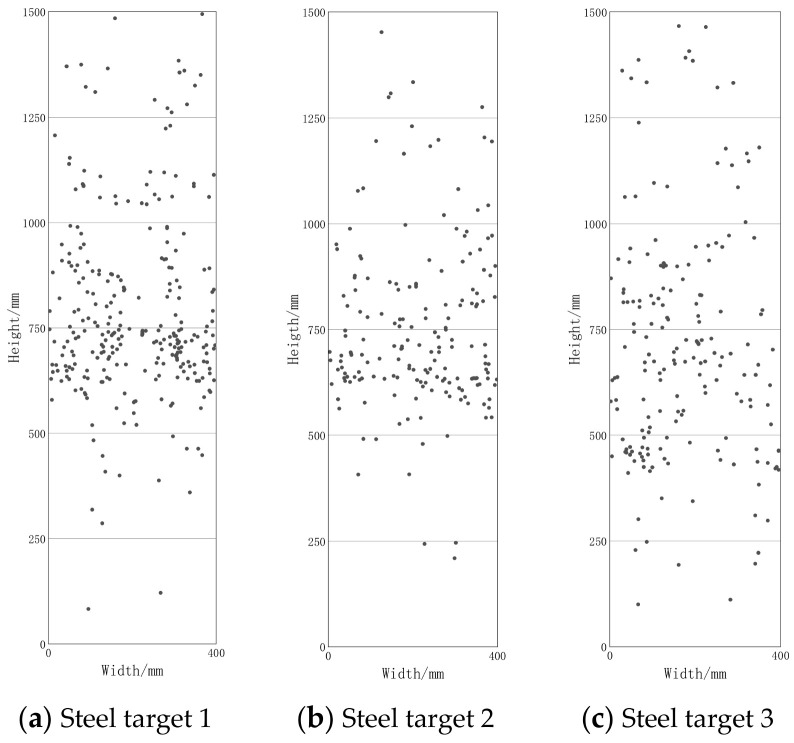
Landing position of fragments on steel targets after explosion.

The landing position of fragments on the steel targets after the explosion is shown in Figure 5. On the steel target 1 which was closest to the warhead, most of the fragments were located in the center area of the target. As the targets moved further away to target 2 and 3, the distribution of fragments became more dispersed. It was also observed that the dimples in the center area of the steel target had greater depth compared with dimples at both ends, which demonstrates that the fragments striking the center area had higher impact velocities compared with those striking the end areas. This is consistent with the observations made with high-speed photography.

In the ballistic gun test, the tungsten fragments with various impact velocities formed dimples of differing sizes on the steel target. The diameter of the dimples was carefully measured and used to calculate the depth of the dimple. The results are shown in Table 1.

In Table 1, *v_b_* is the velocity of a prefabricated fragment, and *h*_0_ is the penetration depth. Using the test results, the De Marre formula is modified to show the relationship between the fragment velocity and the penetration depth:

(1) $$v_{b}=4135\frac{d^{0.937}\cdot h_{0}^{0.563}}{m_{f}{}^{0.5}\cos\omega }$$

where *d* is the prefabricated fragment diameter, and *ω* is the angle between the movement direction of the prefabricated fragment and the normal direction of the target plate. The formula also includes the mass in kilograms, length in decimeters, and velocity in meters per second.

## 4. Discussion

### 4.1. Classic Models for Fragment Velocity and Dispersion Calculation

Gurney assumed that the axial fragments fly out at the same velocity and proposed a famous formula based on energy conservation law [8], which can be expressed as:

(2) $$v_{0}=\sqrt{2E}\sqrt{\frac{C}{M+0.5C}}$$

where $\sqrt{2E}$ is the Gurney constant, *C* is the charge mass, *M* is the shell mass, and *v*_0_ is the Gurney velocity.

However, the Gurney formula does not consider the effect of the sparse waves incoming from the explosion initiation end and the explosive termination end on the initial velocity of the fragment. Therefore, researchers established modified formulas according to the Gurney formula. Based on experimental data, Huang [11] introduced the correction factor into the Gurney formula to describe the axial distribution of cylindrical warhead fragments at the endpoint, which can be expressed as:

(3) $$v_{f}(x)=v_{0}[1-0.361e^{-1.111x/(2r_{0})}][1-0.192e^{-3.03(L-x)/(2r_{0})}]$$

where *x* is the axial distance between the fragment and the explosion initiation end, *r*_0_ is the charge radius, *v_f_* is the initial velocity of the fragment, and *L* is the charge length.

Since the energy released by the explosive cannot be fully utilized by the fragments, to describe the law of distribution of explosive energy along the axial, Randers-Pehrson [12] proposed a method dependent on the axial proportional coefficient to correct the loading mass along the axial direction, which can be expressed as:

(4) $$f(x)=1-{\left\{1-\min[x/(2r_{0}),1,(L-x)/r_{0}]\right\}}^{2}$$

Taking the coefficient into Equation (1), the initial velocity of fragment is shown as:

(5) $$v_{f}(x)=\sqrt{2E}\sqrt{\frac{Cf(x)}{M+0.5Cf(x)}}$$

To describe the initial direction angle of the fragments, Taylor [13] calculated the direction angle of the cylindrical warhead based on the action law of the detonation products on the shell, called the Taylor formula, which is shown as:

(6) $$\alpha (x)=90^{\circ }-\arcsin[\frac{v_{f}(x)}{2D}\cos\theta_{1}]$$

where *θ*_1_ is the angle between the detonation front and the normal to the explosive/metal interface, *D* is the detonation velocity, and *α* is the initial direction angle of the fragment.

Neither the Gurney formula nor the Taylor formula considers the effect of the sparse waves incoming from the explosion initiation end and the explosive termination end on the initial velocity of the fragment. Therefore, researchers [14] introduced the warhead structure parameters into the formula to calculate the initial direction angle, which can be expressed as:

(7) $$\alpha (x)=90^{\circ }-K{\left[\frac{df(x)}{dx}\right]}^{2}\sqrt{\frac{M}{C}+0.5}-\arcsin[\frac{v_{f}(x)}{2D}\cos\theta_{1}]$$

where the coefficient *K* is 1.295 at the explosion initiation end and −6.315 at the explosion termination end, respectively, of the blast-fragmentation warhead.

The above method only discusses the acceleration process of the fragment in the “detonation wave acceleration stage” and the “detonation wave product acceleration stage” [15], while the effect of the interaction between the layers on the fragments initial parameters is ignored [16]. Meanwhile, the difference in the fragments’ initial parameters along the axis [17] and the applicability to the initial parameters of the double-layer prefabricated fragments [18,19,20,21] is not considered.

### 4.2. Three-Stage Detonation Drive Model along the Axial Distribution

In this study, the acceleration process of double-layer prefabricated fragments is divided into three stages. During the “detonation wave acceleration stage”, the detonation wave spreads in the detonation product after the explosion. The detonation wave obliquely incident to the interface of the lining medium forms the reflection wave, and the transmission wave travels from the detonation product to the lining medium. The liner collides with the inner layer of prefabricated fragments at high velocity. The process of the inner layer of prefabricated fragments acting on the outer layer of prefabricated fragments is called the “metal–medium interaction stage”. This paper only discusses the first interaction between the various metal mediums. The “detonation product acceleration stage” is regarded as the acceleration process of the detonation product pressure on the prefabricated fragment based on the first two acceleration stages.

**Figure 6:**
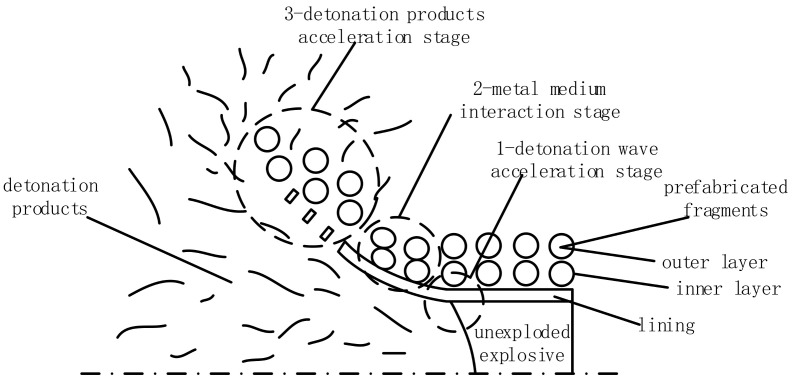
Three-stage detonation driving model of double-layer prefabricated fragments.

It is believed that the “detonation wave acceleration stage” and “metal–medium interaction stage” are completed instantly as the detonation wave and shock wave transmit in the medium with high velocity. Therefore, the “detonation wave acceleration stage” and the “metal–medium interaction stage” are independent of each other,

The “detonation product acceleration stage” is the main stage when prefabricated fragments are accelerated. It is believed that the “detonation product acceleration stage” occurs after the above two acceleration stages, and the initial velocity of prefabricated fragments is the result of the above three stages acting together. A diagram of each acceleration stage is shown in Figure 6.

#### 4.2.1. Detonation Wave Acceleration Stage

**Figure 7:**
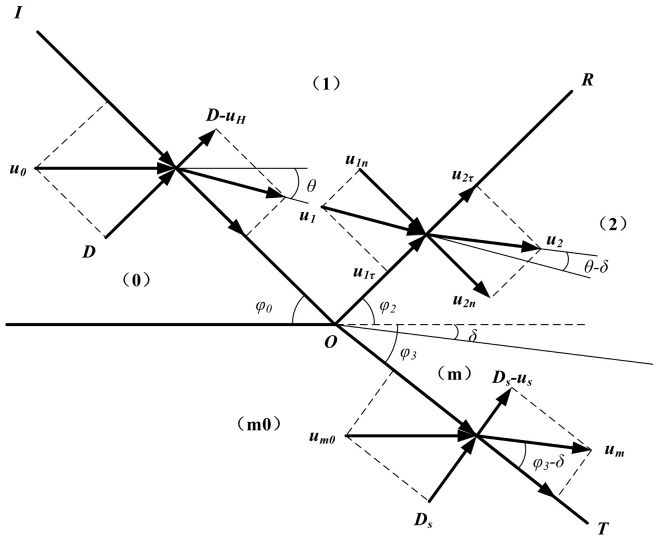
Normal oblique reflection of detonation wave on lining surface.

The detonation wave acceleration phase can be solved via the detonation wave oblique incidence theory. The detonation wave oblique incident model is established by taking the coordinate origin at the point where the detonation wave contacts the medium interface, as shown in Figure 7. *U*_0_ is the velocity component of detonation velocity D along the direction of the metal wall; *U_H_* is the velocity difference of medium on both sides of the OI interface; *U_i_* is the flow velocity of medium in region (*i*); *U_i_n* is the velocity component of *U_i_* normal to the oblique reflected shock wave front; *U_i_t* is the velocity component of *U_i_* along the oblique reflected shock wave front; *Ds* is the velocity of the medium passing through the OT interface and *u_m_* is the velocity component of *Ds* along the metal wall.

In Figure 7, Area (0) is unexploded explosive, Area (1) is the detonation product area behind the oblique detonation wave, Area (2) is the detonation product area behind the reflecting shock wave, Area (m0) is the initial media area, and Area (m) is the area where the medium is disturbed behind the oblique transmission of the shock wave. OI is an oblique detonation wave front. The angle between OI and the contact medium is *φ*_0_. OR is the wavefront of the oblique reflected shock wave in the detonation product. The angle between OR and the initial interface of the media is *φ*_2_. OT is the oblique transmission shock wave front in the medium. The angle between OT and the initial interface of the media is *φ*_3_. The medium is deformed under the action of the detonation products. The angle between the interface and the initial boundary of the media is *δ*. The folding angle *θ* in the model and the Mach number of the region (1) can be shown as [22]:

(8) $$\tan\theta =\frac{\tan\varphi_{0}}{1+k(1+\tan^{2}\varphi_{0})}$$

(9) $$M_{1}=\sqrt{1+{\left(\frac{k+1}{k}\right)}^{2}\cot^{2}\varphi_{0}}$$

where *k* is the isentropic index. According to the geometric relationship of the model, C-J theory [23], the impact compression law of material is expressed as:

(10) $$\sin^{2}(\varphi_{2}+\theta )=\frac{1}{2kM_{1}^{2}}\left[\frac{\rho_{m0}{\left(k+1\right)}^{2}\sin\varphi_{3}}{\rho_{0}b_{m}\sin\varphi_{0}}(\frac{\sin\varphi_{3}}{\sin\varphi_{0}}-\frac{a_{m}}{D})+k-1\right]$$

(11) $$\begin{array}{l} \frac{(k-1)\sin(\varphi_{2}+\theta )-(k+1)\cos(\varphi_{2}+\theta )\tan\varphi_{2}+\frac{2}{M_{1}^{2}\sin(\varphi_{2}+\theta )}}{(k-1)\sin(\varphi_{2}+\theta )\tan\varphi_{2}+(k+1)\cos(\varphi_{2}+\theta )+\frac{2\tan\varphi_{2}}{M_{1}^{2}\sin(\varphi_{2}+\theta )}}\\ =\frac{(1-\frac{a_{m}\sin\varphi_{0}}{D\sin\varphi_{3}})\tan\varphi_{3}}{b_{m}+(b_{m}-1+\frac{a_{m}\sin\varphi_{0}}{D\sin\varphi_{3}})\tan^{2}\varphi_{3}} \end{array}$$

where *ρ*_0_ is the charge density, *ρ_m_*_0_ is the initial density of the lining medium, and *a_m_* and *b_m_* are the Hugoniot parameters of the lining medium. The joint solution of the above two equations results in *φ*_2_ and *φ*_3_. The parameters of media behind the shock wave can be shown as:

(12) $$\rho_{m}=\frac{\rho_{m0}}{1-\frac{1}{b_{m}}(1-\frac{a_{m}\sin\varphi_{0}}{D\sin\varphi_{3}})}$$

(13) $$p_{m}=\frac{\rho_{m0}D^{2}\sin\varphi_{3}}{b_{m}\sin\varphi_{0}}(\frac{\sin\varphi_{3}}{\sin\varphi_{0}}-\frac{a_{m}}{D})$$

(14) $$tg(\varphi_{3}-\delta )=\frac{\rho_{m0}}{\rho_{m}}tg\varphi_{3}$$

(15) $$u_{s}=\frac{D\sin\varphi_{3}}{\sin\varphi_{0}}-\frac{u_{m}}{\sin(\varphi_{3}-\delta )}$$

where *u_m_* is the tangential point velocity of the mass in the lining medium, *ρ_m_* is the lining medium density behind the oblique transmission shock wave, *p_m_* is the lining medium pressure behind the oblique transmission shock wave, and *u_s_* is the lining medium velocity of the shock wave after the oblique transmission.

#### 4.2.2. Metal–Medium Interaction Stage

Shock waves are generated in the lining medium and all layers of the prefabricated fragments to change the motion of the medium when the lining works with the double-layer prefabricated fragments. The equations are obtained according to the conservation equations of the medium before and after the collision, as well as the boundary conditions when considering the collision of the lining with the inner layer of prefabricated fragments:

(16) $$p_{me}-p_{m}=\rho_{m}[a_{m}+b_{m}(u_{s}-u_{se})](u_{s}-u_{se})$$

(17) $$p_{2}-p_{20}=\rho_{20}[a_{2}+b_{2}(u_{2}+u_{20})](u_{2}-u_{20})$$

(18) $$p_{me}=p_{2}$$

(19) $$u_{se}=u_{2}$$

where *u*_20_ is the velocity of the inner layer of prefabricated fragments before the action of the shock wave, *ρ*_20_ is the density of the inner layer of prefabricated fragments before the action of the shock wave, *p*_20_ is the pressure of the inner layer of prefabricated fragments before the action of the shock wave, *u_se_* is the velocity of the lining medium after the action of the shock wave, *p_me_* is the pressure in the lining medium after the action of the shock wave, *u*_2_ is the velocity of the inner layer of prefabricated fragments after the action of the shock wave, *p*_2_ is the pressure of the inner layer of prefabricated fragments after the action of the shock wave, and *a*_2_ and *b*_2_ are the Hugoniot parameters of the inner layer of prefabricated fragments.

**Figure 8:**
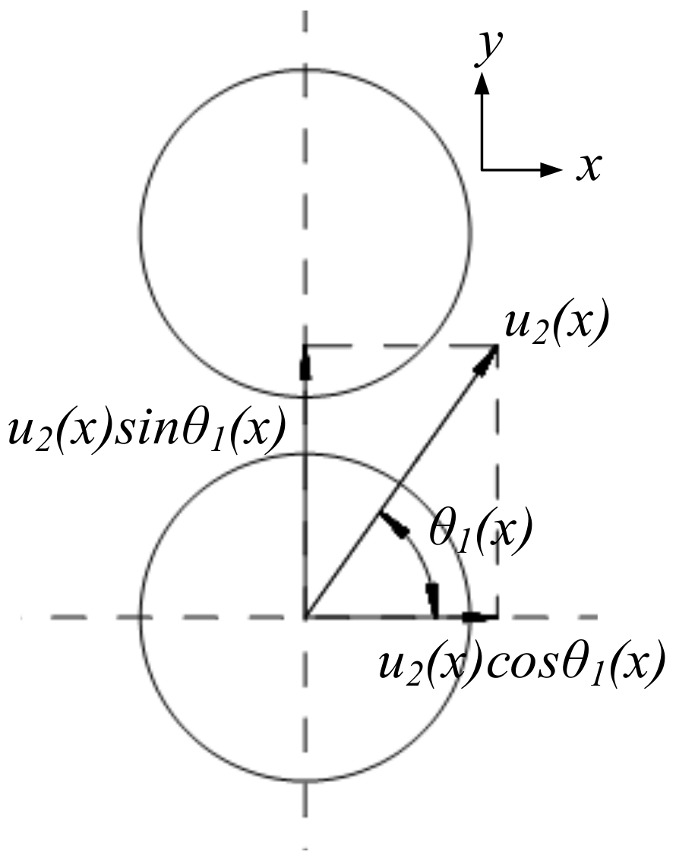
Velocity status before interaction of prefabricated fragments.

The action process of the inner-layer prefabricated fragments on the outer layer prefabricated fragments is discussed based on *u*_2_. The velocity of each layer in the direction of collision becomes half the velocity of the inner layer based on the conservation of momentum, when the outer layer of prefabricated fragments is stationary before the collision and the two layers have the same quality and material. The velocity status of each layer before the collision is shown in Figure 8 when the prefabricated fragments are evenly arranged in the radius direction and the axis direction, where the x-direction is the axial direction of the warhead, the y-direction is the radial direction of the warhead, *θ*_1_ is the angle between the velocity direction and the warhead axis direction after the “metal–medium interaction stage” of the inner layer of prefabricated fragments, the value of *θ*_1_ is π/2-*φ*_3_, and *θ*_1_ is a function of *x*.

**Figure 9:**
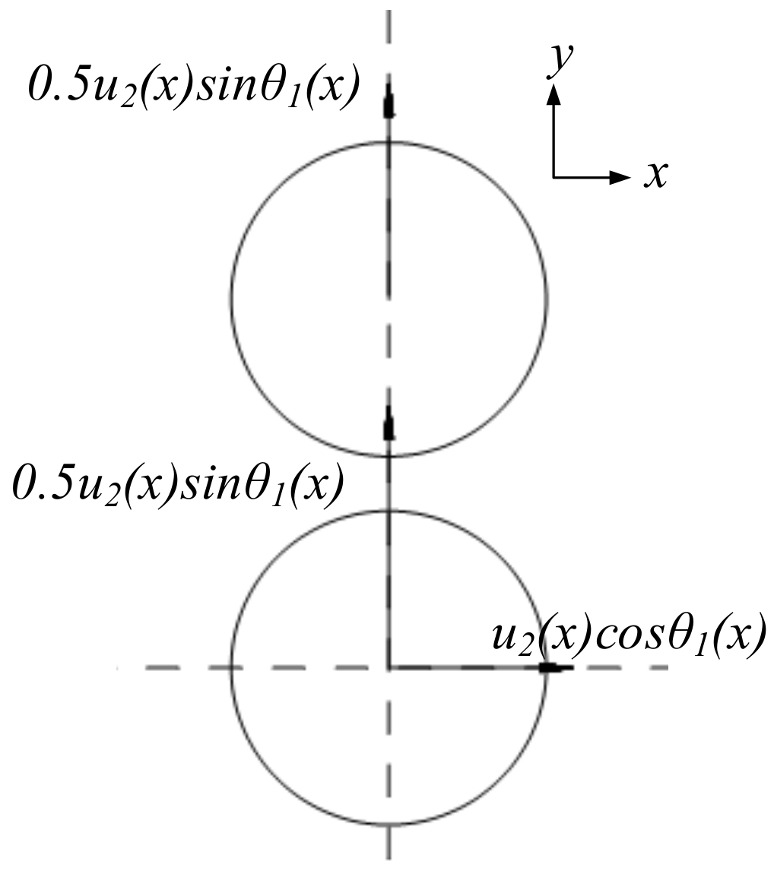
Velocity status after interaction of prefabricated fragments.

The velocity component of the inner layer along the axis of the warhead is *u*_2_(*x*)cos *θ*_1_(*x*), and the velocity component along the radial of the warhead is *u*_2_(*x*)sin *θ*_1_(*x*). The velocity of each layer is shown in Figure 9 after the collision. Therefore, after the “metal–medium interaction stage”, the velocity of the prefabricated fragments is indicated by *V_i_*, and the angle between *V_i_* and the axial direction of the warhead is indicated by *Φ_i_*.

(20) $$V_{i}(x)=\left\{\begin{array}{c} \begin{array}{cc} \sqrt{{\left[u_{2}(x)\cos\theta_{1}(x)\right]}^{2}+{\left[0.5u_{2}(x)\sin\theta_{1}(x)\right]}^{2}} & \left(i=1\right) \end{array}\\ \begin{array}{cc} 0.5u_{2}(x)\sin\theta_{1}(x) & \left(i=2\right) \end{array} \end{array}\right.$$

(21) $$\varphi_{i}(x)=\left\{\begin{array}{c} \begin{array}{cc} \mathrm{atan}[\frac{\tan\theta_{1}(x)}{2}] & \left(i=1\right) \end{array}\\ \begin{array}{cc} \frac{\pi }{2} & \left(i=2\right) \end{array} \end{array}\right.$$

where *i* is the number of the prefabricated fragment layers.

#### 4.2.3. Detonation Products Acceleration Stage

**Figure 10:**
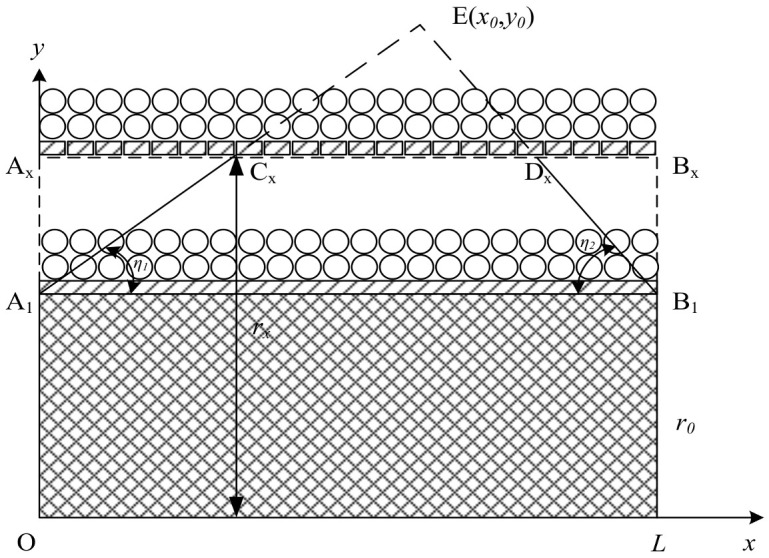
The effect of detonation products on double-layer prefabricated fragments.

The following assumptions are made to analyze the acceleration process of prefabricated fragments: The mass loss of prefabricated fragments is not considered. The explosive product expansion is isentropic. The change of detonation products density caused by the leakage along the axis is ignored. The detonation products are evenly distributed in space, and after the first sparse wave is introduced, the detonation products no longer work on the prefabricated fragments. The model of detonating product action on the double-layer spherical prefabricated fragments is shown in Figure 10, where OX is the symmetry axis of the explosive, the shaded part is the explosive, and the O point is the detonation point. The explosive has openings at both ends, and the outer layer of the explosive has a lining. There are two layers of spherical prefabricated fragments outside the lining, and outside the prefabricated fragments is air. There is no shell to block the outward movement of the fragments.

After the explosive has exploded at point O, there are sparse wave afferent detonation products in the A_1_C_x_ direction at the explosion initiation end, and sparse wave afferent detonation products in the B_1_D_x_ direction at the explosion termination end. The angle between the A_1_C_x_ direction and axial direction is *η*_1_; the angle between the B_1_D_x_ direction and axial direction is *η*_2_; A_1_C_x_ and B_1_D_x_ intersect at the point E with the abscissa *x_0_* and the ordinate *y*_0_. The detonation products in regions A_1_A_x_C_x_ and B_1_B_x_D_x_ no longer work on the prefabricated fragments when the detonation product expands to A_x_B_x_.

After the explosion, the detonation products diffuse outward in a circumferential direction [4,9]. The change in detonation products density caused by the leakage along the axis is ignored. The expression of the detonation product density *ρ_x_* at any time is shown as:

(22) $$\rho_{x}=\rho_{0}{\left(\frac{r_{0}}{r_{x}}\right)}^{2}$$

where *r_x_* is the distance between the detonation product interface and the axis of the warhead, and according to the iso entropy equation, the detonation product pressure at A_x_B_x_ can be shown as:

(23) $$p_{x}=p_{H}{\left(\frac{\rho_{x}}{\rho_{H}}\right)}^{k}=\frac{\rho_{0}D^{2}}{k+1}{\left[\frac{k\rho_{x}}{(k+1)\rho_{0}}\right]}^{k}=\frac{\rho_{0}D^{2}}{k+1}{\left[\frac{kr_{0}^{2}}{(k+1)r_{x}^{2}}\right]}^{k}$$

where *p_H_* and *ρ_H_* are the C-J parameters. *r_max_* is the working radius of the detonation product, which can be shown as:

(24) $$r_{\max}=\left\{\begin{array}{cc} k_{1,i}x+k_{r1,i}r_{0} & \left(x\leq x_{0}\right)\\ k_{2,i}(L-x)+k_{r2,i}r_{0} & \left(x>x_{0}\right) \end{array}\right.$$

where *k*_1,*i*_ and *k*_2,*i*_ represent the tangent value of the angles *η*_1_ and *η*_2_ of the sparse wave in layer *i*, and *k_r_*_1,*i*_ and *k_r_*_2,*i*_ are the correction coefficients of the initiation end radius and the termination end radius of the prefabricated fragments of layer *i* to correct the boundary explosive dimensions [9]. Equation (24) reflects the process of the sparse wave introducing detonation products in the three-stage mode, where coefficients *k*_1,*I*_ and *k*_2,*i*_ represent the slope of the sparse wave introducing detonation products, reflecting the influence of the sparse wave on the overall velocity of the prefabricated fragments. Their value increases with increasing work distance of the detonation products on the prefabricated fragments, which leads to a larger overall velocity of the prefabricated fragments. Therefore, the kinetic energy increment of a single prefabricated fragment can be expressed as:

(25) $$e_{k}=\int_{r_{0}}^{r_{\max}}p_{x}S_{f}dr_{x}$$

where *e_k_* is the kinetic energy increment of a single prefabricated fragment, and *S_f_* is the effective area of a single preformed fragment. Solving the integral, the kinetic energy increment can be shown as:

(26) $$e_{k}(x)=\frac{\rho_{0}D^{2}S_{f}}{\left(1-2k)(k+1\right)}{\left[\frac{r_{0}^{2}k}{k+1}\right]}^{k}[r_{\max}^{1-2k}(x)-r_{0}^{1-2k}]$$

According to the laws of energy conservation, combining Equation (24) with Equation (26), the work performed by the detonation products on the prefabricated fragments can be obtained. Afterwards, by adding this work to the initial velocity obtained from the first two stages of the “three-stage model”, the final initial velocity, *v_i_*, of the prefabricated fragments can be obtained as:

(27) $$v_{i}(x)=\sqrt{V_{i}^{2}(x)+\frac{\xi_{i}e_{k}(x)}{m_{f}}}$$

where *m_f_* is the mass of the prefabricated fragment. In the model, the influence of explosive mass and energy leakage is taken into account in the correction coefficient *ξ_i_* [24]. The axial distribution model of the initial velocity of the double-layer prefabricated fragments can be shown as:

(28) $$v_{i}(x)=\left\{\begin{array}{cc} \sqrt{V_{i}^{2}(x)+\frac{\xi_{i}}{m_{f}}\frac{\rho_{0}D^{2}S_{f}}{\left(1-2k)(k+1\right)}{\left[\frac{r_{0}^{2}k}{k+1}\right]}^{k}[{\left(k_{1,i}x+k_{r1,i}r_{0}\right)}^{1-2k}-r_{0}^{1-2k}]} & \left(x\leq x_{0}\right)\\ \sqrt{V_{i}^{2}(x)+\frac{\xi_{i}}{m_{f}}\frac{\rho_{0}D^{2}S_{f}}{\left(1-2k)(k+1\right)}{\left[\frac{r_{0}^{2}k}{k+1}\right]}^{k}[{\left(k_{2,i}L-k_{2,i}x+k_{r2,i}r_{0}\right)}^{1-2k}-r_{0}^{1-2k}]} & \left(x>x_{0}\right) \end{array}\right.$$

For the above derivation process, each coefficient has its own physical meaning. For *k*_1,*i*_ and *k*_2,*i*_, *i* represents the influence of sparse waves on the fragments at the initiation and termination ends of the *i*-th layer explosion. For *kr*_1,*i*_ and *kr*_2,*i*_, *i* represents the influence of the explosives on the initiation and termination fragments of the *i*-th layer explosion, and *ξ_i_* represents the utilization of the detonation energy by the *i*-th layer fragments.

#### 4.2.4. Direction Angle of Double-Layer Prefabricated Fragments

**Figure 11:**
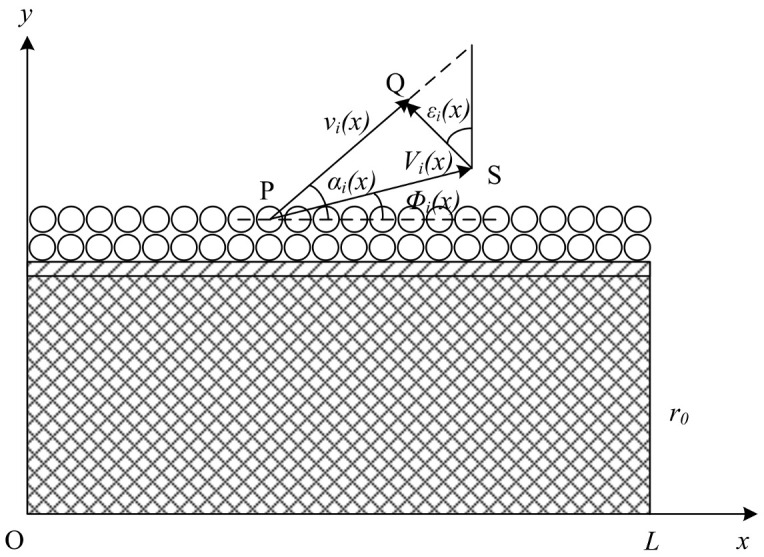
Model of dispersion angle of prefabricated fragments.

The direction angle model of the double-layer spherical prefabricated fragment is shown in Figure 11. The initial velocity obtained by the prefabricated fragments in the first stage is *V_i_*(*x*), and the scattering direction angle of the fragments is *Φ_i_*(*x*). The initial velocity obtained by the fragments after three acceleration processes is *v_i_*(*x*).

In the model, *i* is the direction angle of the prefabricated fragment in layer *i*, *v_i_* is located along the PQ direction, and *ε_i_* is the angle between the SQ direction and the axis direction. An equation can be established by using the velocity triangle:

(29) $$\frac{v_{i}(x)}{\sin[\frac{\pi }{2}-\varepsilon_{i}(x)+\varphi_{i}(x)]}=\frac{V_{i}(x)}{\sin[\frac{\pi }{2}-\alpha_{i}(x)+\varepsilon_{i}(x)]}$$

and the direction angle is:

(30) $$\alpha_{i}(x)=\arccos\{\frac{V_{i}(x)}{v_{i}(x)}\cos[\varphi_{i}(x)-\varepsilon_{i}(x)]\}+\varepsilon_{i}(x)$$

Therefore, the axial distribution model of the direction angle of double-layer prefabricated fragments can be expressed as:

(31) $$\alpha_{i}(x)=\left\{\begin{array}{cc} \arccos\{{\left[\frac{\xi_{1}e_{k}(x)}{m_{f}V_{1}^{2}(x)}+1\right]}^{-0.5}\cos[\mathrm{atan}(\frac{\tan\theta_{1}(x)}{2})-\varepsilon (x)]\}+\varepsilon (x) & \left(i=1\right)\\ \arccos\{{\left[\frac{\xi_{2}e_{k}(x)}{m_{f}V_{2}^{2}(x)}+1\right]}^{-0.5}\sin\varepsilon (x)\}+\varepsilon (x) & \left(i=2\right) \end{array}\right.$$

where *ε_i_* is described by using the inverse proportional function:

(32) $$\varepsilon_{i}(x)=\frac{1}{k_{3,i}\frac{x}{L}+k_{4,i}}-k_{5,i}$$

where *ε_i_* is the radian system, and *k*_3,*i*_, *k*_4,*i*_ and *k*_5,*i*_ are the correction coefficients.

### 4.3. Simulation Results of the Three-Stage Detonation Drive Model

The finite element simulation results were used to obtain the correction coefficient in Equations (28) and (31). The quarter finite element model of the double-layer prefabricated fragments warhead was established. The mechanical properties of the lining materials are described by the Johnson–Cook constitutive model [25], and the mechanical properties of the prefabricated fragment material are described by the Elastic model. The loading radius of the warhead is 38 mm; the loading length is 100 mm; the diameter of the prefabricated fragment is 2.5 mm, and the thickness of the steel lining is 1.3 mm. The parameters of the explosives are shown in Table 2, and the parameters of the metallic materials are shown in Table 3.

**Figure 12:**
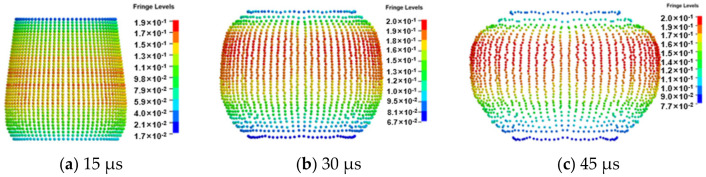
Dispersion of prefabricated fragments.

The dispersion of the double-layer prefabricated fragments obtained by finite element simulation is shown in Figure 12. The explosive detonates, and the detonation wave propagates from bottom to top. The fragments undergo detonation waves, interactions between fragments, and acceleration of detonation products to obtain velocity. At 15 μs, the detonation wave is transmitted to the upper end of the charge, and the interaction between the fragments behind the wavefront is complete. The detonation products have performed work on the prefabricated fragments at the lower end of the charge, and the sparse wave has also been transmitted to the detonation products. Therefore, the initial velocity of the upper fragment is low, while the initial velocity of the lower fragment is high. The fragments close to the initiation point are greatly affected by the sparse wave, so the velocity of these fragments is low. At 30 μs, the acceleration process of the detonation wave on the prefabricated fragments and the interaction between the fragments are complete. The effect of the detonation product on the upper fragment has just begun, so the initial velocity of the upper fragment is smaller than that of the lower one. At 45 μs, the acceleration effect of the detonation product on the fragments is almost complete, and at this time, the influence of sparse waves on the velocity of the fragments at both ends is very obvious.

The values of the coefficients in the model (27) and model (30) fitted by the average value of the simulation velocity of each column of prefabricated fragments are shown in Table 4. The coefficients in the model (27) and model (30), whose values were obtained via fitting, are listed in the table.

It is found that the energy utilization rate of detonation products acting on the fragments from the inner layer of fragments is 69%, while the rate for the fragments from the outer layer of fragments is 56%. Therefore, *k*_1,1_ is smaller than *k*_1,2_, and *k*_2,1_ is smaller than *k*_2,2_. The deceleration effect of sparse waves on the outer layer of fragments is smaller than that on the inner layer.

As shown in Table 4, the incoming angle at the initiation end of each layer is smaller than that at the termination end. The integral radius *r_max_* of the initiation end of each layer is less than the termination end, indicating that the workability of explosives on prefabricated fragments is proportional to the distance between the fragments and the initiation end. The energy utilization rate of the inner layer is higher than that of the outer layer after considering the mass of the explosive and the leakage of the detonation products. Negative numbers exist for the angle *ε_i_*, which indicates that as the distance between the prefabricated fragments and the initiation point increases, *ε_i_* gradually decreases to 0 and increases afterwards.

**Figure 13:**
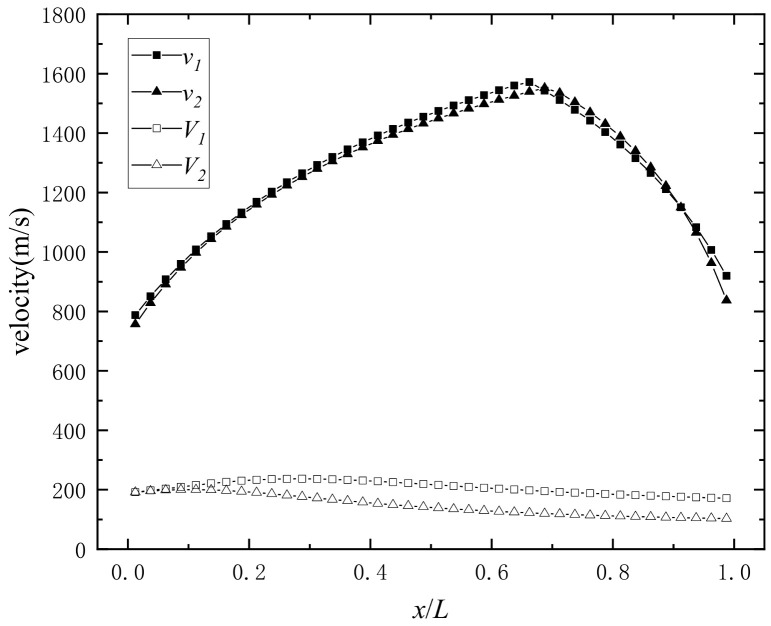
The axial distribution of the initial velocity of prefabricated fragments.

The initial velocity of the double-layer prefabricated fragments obtained from Equation (28) is shown in Figure 13.

In Figure 13, the final initial velocities *v*_1_ and *v*_2_ are similar after the “detonation products acceleration stage”, and the maximum value of *v_i_* is around *x*_0_/*L* = 0.67. As *x* increases, *v_i_* shows increasing regularity with decreasing slope when *x* < *x*_0_, and *v_i_* shows decreasing regularity when *x* > *x*_0_. The *V_i_* of the prefabricated fragments before the “detonation product acceleration stage” shows a first increasing and then decreasing pattern. The ratio of *v_i_* to *V_i_* is between 4 and 13; therefore, the energy obtained by the prefabricated fragment in the “detonation products acceleration stage” and the energy obtained by the prefabricated fragment in the “metal–medium interaction stage” is between 15 and 168, which indicates that the “detonation product acceleration stage” is the main stage at which the initial velocity of the prefabricated fragments is obtained. This shows that the acceleration ability of the detonation products to the prefabricated fragments is much greater than that of the detonation wave and the shock wave [26].

**Figure 14:**
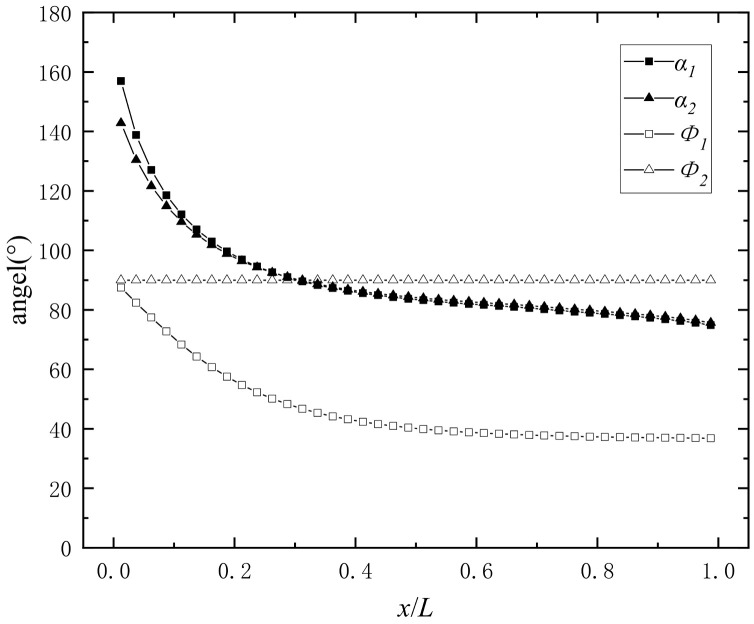
The axial distribution of the divergence angle of prefabricated fragments.

The correction coefficient is brought into Equation (31) and the initial direction angle of each column is obtained as shown in Figure 14.

In Figure 14, the values *α*_1_ and *α*_2_ of the direction angle of the two layers after the “detonation products acceleration stage” almost coincide. As x increases, *α_i_* is quickly reduced to *x*/*L* = 0.2 and then slowly to *x*/*L* = 1. The value of *α_i_* is greater than 90 at the initiation end, indicating that some of the prefabricated fragments near the initiation end disperse in the opposite direction of the detonation wave. The scatter direction *Φ*_1_ of the inner layer is similar to the direction angle *α*_1_, and *α*_1_ > *Φ*_1_, but the difference between *Φ*_2_ and *α*_2_ decreases with increasing *x*, and increases inversely after decreasing to zero.

### 4.4. Comparison between Experimental Results and the Three-Stage Detonation Drive Model Calculation

**Figure 15:**
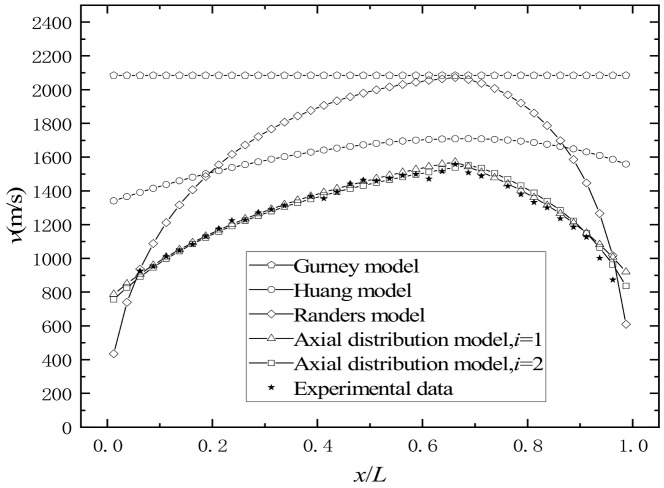
Comparison diagram of the initial velocity distribution of prefabricated fragments along the axial direction.

The penetration depth of the prefabricated fragments on the steel target was measured, and the results of the initial velocity distribution along the axis of the prefabricated fragments are shown in Figure 15. The experiment cannot distinguish which layer of the prefabricated fragments hits the target, so the experimental results show the average velocity of the prefabricated fragments.

With the increase in the distance between the prefabricated fragment and the initiation end, the initial velocity of the prefabricated fragment increases first and then decreases, and the maximum velocity of 1557 m/s appears at around *x*/*L* = 0.66. The calculation results of Equations (3), (4) and (28) all show a trend of initial increase followed by a decrease with increasing distance, and the maximum value appears at around *x*/*L* = 0.67, which is consistent with the test results. Equation (2) cannot reflect the pattern of velocity change with the axial direction. Equation (3) has a maximum error greater than 900 m/s, and Equation (4) is only similar to the test results near the maximum velocity. In conclusion, Equations (2)–(4) show large errors when used for the calculation of the initial velocity of the double-layer prefabricated fragments. The calculation results of the three-stage detonation driving model almost coincide with the test results.

**Figure 16:**
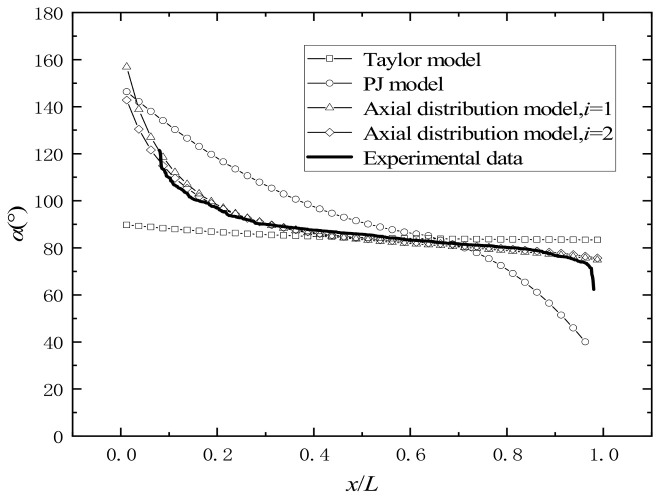
Comparison diagram of the axial distribution of the dispersion direction angle of prefabricated fragments.

The results of the initial direction angle of the prefabricated fragment distribution along the axis of the prefabricated fragments are shown in Figure 16.

With the increase in the distance between the prefabricated fragment and the initiation end, the initial direction angle of the prefabricated fragment decreases to around 90º, then decreases steadily. Afterwards, it shows a trend of rapid decrease again near the termination end of the explosion. The calculation results of Equation (6) show a trend of initial decrease and then increase, which is inconsistent with the test results. The calculation results of Equations (7) and (31) are similar to the test results. The calculated results of Equation (7) differ from the test results at the explosion termination end of the warhead. In conclusion, Equations (6) and (7) cannot be used to calculate the angle of the fragments. The calculation results of the three-stage detonation driving model have the smallest error, and the calculation results almost coincide with the test results.

### 4.5. Remarks about the Three-Stage Detonation Drive Model and Future Work

For the initial velocity model, the Gurney model did not consider the axial distribution of velocity or the phenomenon of reduced energy utilization due to the effect of detonation products on multi-layer fragments. Therefore, the Gurney model results in a higher velocity and only one value. The Huang model and Randers model are models obtained from experiments, where the values of each coefficient are fitted based on the experimental results of a specific warhead (which are all single-layer fragments), and are not applicable to the double-layer fragment structure of the warhead studied in this research.

Considering the scattering direction angle, the Taylor model did not consider the effect of lateral coefficient waves on the scattering direction angle of fragments, and the calculated results were all around 90°, which is inconsistent with the actual situation. The coefficients in the PJ model were obtained based on specific warhead results, and the results of the coefficients are not applicable to the double-layer fragment structure of the warhead studied here.

The three-stage driving model proposed in this article calculates the initial velocity and dispersion direction angle of fragments. It is suitable for the dual-layer fragment structure of the warhead studied in this article, and the calculation results fit well with the experimental results.

In the derivation process, it is shown that when deriving the “metal–medium interaction stage” of fragments in various arrangement modes, due to the effect of the inner-lining medium on the inner-layer fragments, the medium state of the inner-layer fragments is different from that of the outer-layer fragments. However, in order to simplify the calculation, this study assumes that the medium state of the inner- and outer-layer fragments is the same at this time, which causes errors in the calculation results. In order to simplify the calculation model, the fragment initial velocity theory calculation model introduces coefficients for modifying the shape of the warhead, correcting the sparse wave input angle and energy utilization rate. Although these coefficients have their own physical meanings, their specific physical calculation methods need to be further improved.

## 5. Conclusions

A three-stage detonation driving model was built by considering the metal–medium interaction stage between the layers of fragments, and a static explosion test was performed. The following conclusions can be obtained:

(1) The energy of detonation products is enormous; therefore, the “detonation product acceleration stage” is the main stage in the three-stage detonation driving model of double-layer prefabricated fragments.
(2) The De Marre formula can accurately describe the relationship between the initial velocity of prefabricated fragments and the penetration depth on the target.
(3) With the increase in the distance between the prefabricated fragments and the initiation end, the initial velocity of the prefabricated fragment increases first and then decreases, while the maximum initial velocity of the prefabricated fragment appears at around *x*/*L* = 0.66.
(4) The obstruction of the outer-layer fragment during the flow of detonation products reduces detonation products leaking from the inner-layer fragment area. The outer-layer fragments are surrounded by air, and there is no obvious obstruction to the outward flow of detonation products from the outer-layer fragments. Therefore, the energy utilization rate of the inner-layer fragment is 69%, which is higher than the value for the outer-layer fragments (56%).
(5) The initial parameters of double-layer prefabricated fragments calculated from the three-stage detonation driving model are in good agreement with the test results. The model can provide theoretical support and a design scheme for the initial parameter design of double-layer prefabricated fragments warheads.

## Figures and Tables

**Table 1:** Penetration depth of tungsten fragments at various impact velocities.

Test	*h*_0_ (dm)	*v_b_* (m/s)
1	0.0113	870
2	0.0198	1199
3	0.0262	1394
4	0.0321	1570

**Table 2:** Parameters of the JWL equation of state for explosives.

Explosive	ρ_0_/(g·cm^−3^)	D/(m·s^−1^)	P_CJ_/GPa	A/GPa	B/GPa	R_1_	R_2_	Ω	E_0_/GPa
8701	1.695	8450	29.66	854.5	20.49	4.6	1.35	0.25	9.5

**Table 3:** The parameters of the metal materials.

Parameters	45 Carbon Steel	Tungsten Alloy
density/(g·cm^−3^)	7.83	17.6
shear modulus/GPa	77.0	136
Young’s modulus/GPa	200	350
Poisson ratio	0.32	0.286
A	792	/
B	510	/
N	0.26	/
C	0.014	/
M	1.03	/
*a_m_*/(m/s)	3574	/
*b_m_*	1.92	/
*a*_2_/(m/s)	/	4029
*b*_2_	/	1.237

**Table 4:** Correction coefficient in three-stage detonation driving model.

Model	Parameters	Values
The axial distribution model of the initial velocity of double-layer prefabricated fragments	*k*_1,1_	0.0805
	*k*_2,1_	0.1450
	*k_r_*_1,1_	1.0251
	*k_r_*_2,1_	1.0354
	*ξ*_1_	1.3887
	*k*_1,2_	0.1135
	*k*_2,2_	0.2510
	*k_r_*_1,2_	1.0245
	*k_r_*_2,2_	1.0424
	*ξ*_2_	1.1207
The axial distribution model of the direction angle of double-layer prefabricated fragments	*k*_3,1_	5.9252
	*k*_4,1_	0.5143
	*k*_5,1_	0.2878
	*k*_3,2_	5.7807
	*k*_4,2_	0.6312
	*k*_5,2_	0.2717

## Data Availability

Not applicable.
